# Health Outcomes Associated with Asymptomatic *Toxoplasma gondii* Seropositivity in Young Adults: A Nationwide Matched Cohort Study

**DOI:** 10.3390/microorganisms14040780

**Published:** 2026-03-30

**Authors:** Sarah Israel, Eugene Merzon, Yotam Shenhar, Shai Ashkenazi, Abraham Weizman, Shlomo Vinker, Eli Magen, Ariel Israel

**Affiliations:** 1 Department of Clinical Microbiology and Infectious Diseases, Hadassah-Hebrew University Medical Center, Faculty of Medicine, Hebrew University of Jerusalem, P.O. Box 12000, Jerusalem 9112001, Israel; sarahi@hadassah.org.il; 2 Leumit Research Institute, Leumit Health Services, 23 Sprinzak St., Tel Aviv 6473817, Israelsvinker@leumit.co.il (S.V.); 3 Adelson School of Medicine, Ariel University, Ariel 4070000, Israel; 4 Department of Infectious Diseases, Schneider Children’s Medical Center, 14 Kaplan St., Petah Tikva 4920235, Israel; 5 Research Unit, Geha Mental Health Center, Tel Aviv 6997801, Israel; 6 Felsenstein Medical Research Center, Tel Aviv 6997801, Israel; 7 Sagol School of Neurosciences, Faculty of Medical & Health Sciences, Tel Aviv University, Tel Aviv 6997801, Israel; 8 Medicine A Department, Assuta Ashdod University Medical Center, Derech HaRofe 7, Ashdod 7747629, Israel; allergologycom@gmail.com; 9 Department of Epidemiology and Preventive Medicine, School of Public Health, Gray Faculty of Medical & Health Sciences, Tel Aviv University, Ramat Aviv, Tel Aviv 6997801, Israel

**Keywords:** *Toxoplasma gondii*, delayed pathogenicity, latent infection, bradyzoites, overall mortality, behavioral alteration, risk taking

## Abstract

*Toxoplasma gondii* establishes latent infection in a substantial proportion of the global population, yet the long-term health consequences of this infection remain incompletely characterized. We conducted a retrospective observational matched cohort study using longitudinal electronic health record data from a nationwide integrated healthcare provider, including members aged 18–45 years who underwent routine *Toxoplasma* serologic screening, typically performed in obstetric evaluation, excluding patients with clinical toxoplasmosis, immunosuppression, or HIV. Seropositive individuals were matched 1:1 without replacement to seronegative controls to align demographic, temporal, and socioeconomic variables. Time-to-event associations with predefined medical conditions were evaluated using Cox proportional hazards models with false discovery rate correction. The final cohort included 19,443 seropositive individuals and 19,443 matched controls (96.7% female), with a tight baseline balance of demographic and temporal characteristics. During follow-up, seropositivity was associated with increased risks of tobacco dependence (aHR 1.65), alcohol dependence (2.32), suicide attempt (1.82), motor vehicle accidents (1.22), and work accidents (1.27), as well as multiple infectious conditions, including hepatitis B (1.55), hepatitis C (2.15), and syphilis (2.43), with an overall trend toward increased all-cause mortality (1.32, 95% CI [1.00–1.74]). These findings suggest that asymptomatic *Toxoplasma* infection in young adults is associated with increased long-term behavioral and medical comorbidity.

## 1. Introduction

*Toxoplasma gondii* is one of the most prevalent chronic parasitic infections worldwide, with global seroprevalence exceeding 50–60% in some regions. Global seroprevalence varies widely across regions depending on climate, dietary habits, food preparation practices, and environmental exposure to oocysts [1,2,3,4,5]. Following primary infection, which is typically asymptomatic in immunocompetent individuals, the parasite establishes lifelong tissue cysts, notably within neural and muscular tissues [6,7,8,9]. Although latent infection has traditionally been considered clinically silent in healthy hosts, accumulating experimental and epidemiologic evidence suggests that chronic carriage may be associated with subtle neurobiological and systemic effects [10,11,12,13,14]. The long-term health consequences of this latent state, however, remain incompletely characterized.

Most epidemiologic investigations of *T. gondii* have focused on congenital infection, disease in immunocompromised hosts [3,15], or selected associations, particularly neuropsychiatric outcomes such as schizophrenia, dementia, and cognitive impairment [13,16,17,18,19]. Additional studies have examined outcomes possibly related to behavioral alterations, including traffic accidents, self-directed violence, and suicide attempts [11,20,21,22,23]. However, to our knowledge, no large-scale longitudinal study has systematically evaluated a comprehensive range of medical outcomes among otherwise healthy individuals identified through routine *T. gondii* serologic screening.

In our nationwide healthcare system, *Toxoplasma* serologic testing is routinely performed in women of reproductive age as part of obstetric or preconception evaluation, and occasionally in their partners in the context of medically assisted reproduction, to prevent and mitigate the risks of congenital toxoplasmosis and its related pregnancy complications [3,24,25]. This structured screening framework provides an opportunity to identify largely asymptomatic carriers within a predominantly healthy young adult population and to follow them longitudinally using comprehensive nationwide electronic health record data.

To address this gap, we conducted a retrospective matched cohort study within a nationwide integrated healthcare system to evaluate long-term health outcomes associated with *T. gondii* seropositivity among non-immunocompromised adults aged 18–45 years without clinical toxoplasmosis.

## 2. Methods

### 2.1. Study Design and Data Source

We conducted a retrospective matched cohort study using longitudinal electronic health record data from Leumit Health Services (LHS), a nationwide Israeli integrated healthcare organization serving approximately 740,000 members. A centralized electronic health record system has been in continuous use for more than two decades across all ambulatory care settings. Diagnoses are recorded by treating physicians using a curated diagnostic interface mapped to the International Classification of Diseases (ICD). Laboratory testing is performed in a central accredited laboratory facility using standardized and calibrated procedures. In addition to laboratory results, encoded diagnoses, anthropometric measurements, and medication dispensations, the LHS database includes comprehensive demographic information, including ethnic sector classification (General, Jewish ultra-orthodox, or Arab) and socioeconomic status on a 1–20 scale derived from geocoded residential data, as well as automatic linkage to national mortality registry updates. These centrally curated data are maintained for population-based research through validated extraction and analysis pipelines developed by the Leumit Research Institute and have supported multiple large-scale epidemiologic investigations [26].

#### Study Population and Exposure Definition

Eligible individuals were LHS members aged 18–45 years with documented *T. gondii* serologic testing, most commonly performed as part of obstetric or preconception screening. Participants were required to be actively enrolled in LHS and alive at the time of testing and to have at least two years of continuous membership to ensure adequate longitudinal data for outcome assessment. Individuals with documented immunosuppression, HIV infection, or clinical toxoplasmosis diagnosed up to one year following serologic testing were excluded to restrict the cohort to non-immunocompromised individuals and to avoid inclusion of those tested in the context of suspected acute disease or in whom positive serology reflected an underlying immunosuppressive condition.

Seropositive individuals were defined as those with any recorded positive *Toxoplasma* IgG result, indicating prior exposure. Controls were selected among individuals with negative serology (equivocal results were excluded) and no documented positive result in their medical record. Exposure classification was based solely on serologic status and did not condition on future health outcomes. Because exposure assignment required survival and active membership until the date of serologic testing, both exposed and unexposed individuals satisfied identical survival and enrollment criteria at the time of classification.

### 2.2. Serologic Assay Details

*T. gondii* IgG and IgM antibodies were measured in the central Leumit Health Services laboratory using commercially available chemiluminescent microparticle immunoassays (CMIA) from Abbott Diagnostics, performed on the Alinity i platform (Abbott Laboratories, Abbott Park, IL, USA). Assays were conducted according to the manufacturer’s instructions for use, with calibration and quality control procedures performed routinely in the accredited central laboratory. Serologic interpretation followed manufacturer-defined cutoffs implemented in the laboratory information system: *T. gondii* IgG results were classified as nonreactive (<1.6 IU/mL), equivocal (1.6 to <3.0 IU/mL), or reactive (≥3.0 IU/mL), and IgM results as nonreactive (<0.50 index), equivocal (0.50 to <0.60 index), or reactive (≥0.60 index). Equivocal results were excluded from analysis.

### 2.3. Follow-Up

To minimize immortal time bias, the matching procedure incorporated strict alignment on the calendar date of first electronic health record (EHR) entry and the calendar date of serologic testing, thereby ensuring comparable observation windows and exposure ascertainment periods across groups. The interval between the first EHR entry and serologic testing was balanced between matched individuals. In sensitivity analyses using the date of serologic testing as the time origin, hazard ratio estimates were virtually identical, and no deaths occurred between alternative time origins. These analyses reduce concern for immortal time bias.

### 2.4. Matching Procedure

To ensure a tight balance on demographic characteristics and calendar anchors, we implemented a hybrid matching strategy combining exact matching and propensity score–based nearest-neighbor matching within calipers. Exact matching was required on sex, ethnic sector, current pregnancy status, prior pregnancy history, and infertility diagnosis as recorded in the EHR at the time of serologic testing. Propensity scores were estimated using logistic regression modeling the probability of *Toxoplasma* seropositivity as a function of age at testing, socioeconomic status (1–20 geographically derived scale), calendar test date, and birth date. Nearest-neighbor matching was then performed within a predefined caliper on the logit of the propensity score.

In addition to propensity score distance, strict caliper constraints were imposed on birth date, age at serologic testing, calendar date of first EHR record, calendar date of serologic testing, and socioeconomic status. These simultaneous constraints ensured close alignment in age, calendar time, and exposure opportunity, thereby minimizing secular trend and temporal bias.

Behavioral or metabolic factors such as smoking status, alcohol use, or body mass index were not included in the matching algorithm. These variables may represent potential downstream mediators of infection-related effects rather than baseline determinants of exposure, and conditioning on such variables could introduce overadjustment bias and obscure possible infection-associated differences. In addition, *Toxoplasma* screening in this population was largely performed as part of routine obstetric evaluation and was not triggered by smoking status, BMI, or other lifestyle characteristics. Baseline BMI distributions were similar between groups after demographic matching (Table 1), suggesting that a major anthropometric imbalance was unlikely.

Covariate balance was assessed using standardized mean differences (SMD), with an absolute value below 0.05 prespecified as indicating excellent balance. Post-matching SMDs for key temporal covariates were below 0.02, including year of first EHR record, year of serologic testing, and age at testing, confirming tight calendar alignment.

### 2.5. Outcome Definitions

Outcomes were derived from a predefined structured list of 300 common medical conditions curated by the Leumit Research Institute for epidemiologic studies. Diagnoses were identified using physician-recorded International Classification of Diseases (ICD) codes and mapped through standardized category groupings. Because seropositivity does not provide information regarding the timing of infection acquisition, outcomes were evaluated as incident events relative to structured EHR follow-up, with sensitivity analyses performed at serology testing yielding consistent results for almost all outcomes. All-cause mortality was ascertained through automated linkage with the national population registry.

### 2.6. Statistical Analysis

Time-to-event analyses were conducted using Cox proportional hazards regression models. Hazard ratios were estimated with clustering by matched strata to account for the paired design and with residual adjustment for age at cohort entry, sex, and five-level socioeconomic status category. In sensitivity analyses, models were additionally adjusted for the number of outpatient visits in the year preceding the index date as a proxy for healthcare utilization.

In exploratory enrichment analyses, Fisher’s exact tests were applied, and false discovery rate correction was performed using the Benjamini–Hochberg procedure, with q < 0.05 considered statistically significant.

Continuous variables were compared using independent two-sample t-tests and categorized when required using predefined reproducible algorithms. Missing body mass index (BMI) (~5%) and smoking status (~9%) were similarly distributed between groups and were retained as explicit categories to preserve cohort size and avoid potential bias from complete-case restriction. To mitigate type I error, all models were corrected for multiple comparisons using false discovery rate procedures, and only associations surviving correction are reported.

All analyses were conducted in R version 4.4, with data preprocessing performed using structured analysis pipelines in Python version 3.11. All statistical tests were two-sided.

### 2.7. Ethics and Reporting

The study protocol was approved by the Leumit Health Services Institutional Review Board with waiver of informed consent due to use of de-identified routinely collected data. Reporting followed the STROBE guideline and the RECORD extension for studies using routinely collected health data.

## 3. Results

### 3.1. Cohort Construction

**Figure 1:**
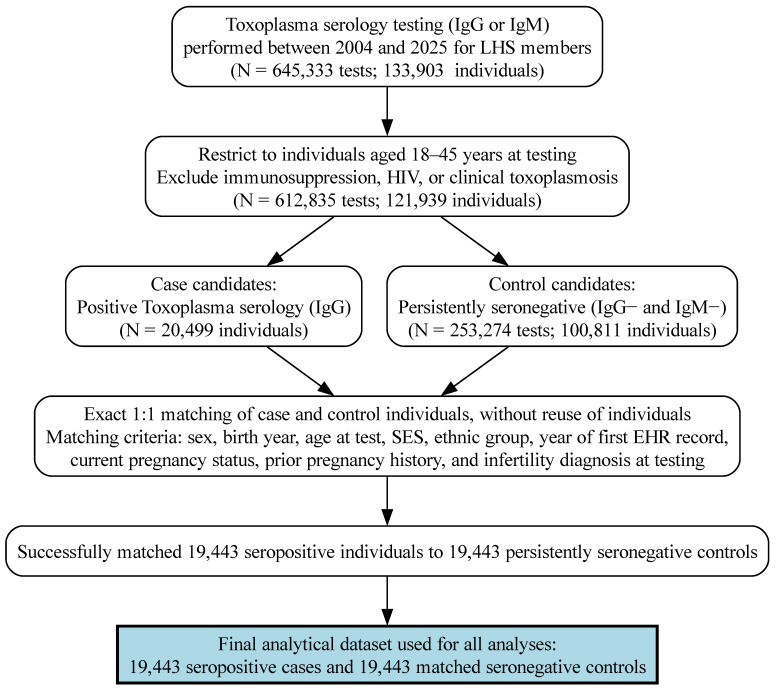
Cohort Construction Flowchart. Flow diagram illustrating identification of the study population from Leumit Health Services electronic health records. Between 2004 and 2025, 645,333 *Toxoplasma* serology tests were performed in 133,903 individuals. After restriction to adults aged 18–45 years at testing and exclusion of immunosuppression, HIV infection, or clinical toxoplasmosis, 121,939 eligible individuals remained. Among these, 20,499 individuals with positive serology were identified as case candidates, and 100,811 persistently seronegative individuals as control candidates. Exact 1:1 matching without replacement was performed based on sex, birth year, age at test, socioeconomic status, ethnic group, year of first EHR record, pregnancy-related variables, and infertility diagnosis at testing. A total of 19,443 seropositive individuals were successfully matched to 19,443 seronegative controls, forming the final analytical cohort of 38,886 individuals.

Between 2004 and 2025, a total of 645,333 *Toxoplasma* serology tests were performed among 133,903 members of Leumit Health Services. After restricting the cohort to individuals aged 18–45 years at the time of testing and excluding those with immunosuppression, HIV infection, or clinically documented toxoplasmosis, 121,939 individuals remained eligible for analysis (Figure 1).

Among these, 20,499 individuals had at least one positive *Toxoplasma* serology (IgG or IgM), and 100,811 individuals were persistently seronegative. Using exact 1:1 matching without replacement, 19,443 seropositive individuals with positive IgG results were successfully matched to 19,443 persistently seronegative controls, yielding a final analytic cohort of 38,886 individuals (Figure 1).

### 3.2. Baseline Characteristics

Baseline characteristics of the matched cohort are presented in Table 1. The study population consisted of young adults, with more than half aged 18–29 years at the index date and over 96% female, reflecting predominantly routine serologic screening of women of reproductive age and, occasionally, their partners in clinical care.

Post-matching balance was assessed using standardized mean differences (SMD). All matched variables demonstrated excellent balance, with absolute SMD values below 0.05, indicating close alignment of demographic and temporal characteristics between groups. Calendar alignment was particularly tight, with identical distributions of index serology year, year of first EHR record, and age at testing. The year of first EHR record averaged 2006 ± 5 in both groups, and the index serology year averaged 2013 ± 6, supporting comparable longitudinal observation windows.

Ethnic sector and socioeconomic status were closely aligned, in accordance with the matching procedure. Clinical characteristics, which were not explicitly constrained during matching, were generally well-balanced at baseline. Mean body mass index was nearly identical between groups, and systolic blood pressure showed minimal difference (SMD 0.033). Smoking status showed a modest increase in current smokers (absolute SMD 0.074), reflecting a small difference in a behavioral variable not included in the matching algorithm.

Overall, the matched seropositive and seronegative cohorts demonstrated excellent comparability for demographic and calendar variables, supporting the comparability of subsequent time-to-event analyses while allowing clinically relevant differences to emerge during follow-up.

### 3.3. Laboratory Measure Comparisons

We compared laboratory parameters at baseline, using measurements obtained prior to or at the index serology test. For nearly all routinely measured tests, including creatinine, estimated glomerular filtration rate, fasting glucose, LDL and HDL cholesterol, albumin-to-creatinine ratio, and hemoglobin, standardized mean differences were small (all <0.05), indicating no material baseline imbalance in common indicators of general health. These findings are consistent with a largely healthy cohort at study entry with predominantly asymptomatic infection status.

In contrast, several immune-related and micronutrient parameters showed small but consistent directional differences of uncertain clinical significance at the time of index testing (Table 2). Seropositive individuals had slightly lower total white blood cell counts (8.51 ± 2.49 vs. 8.78 ± 2.45; SMD −0.107), accompanied by lower neutrophil percentages (66.5 ± 8.8 vs. 67.7 ± 8.6; SMD −0.136), higher lymphocyte percentages (24.3 ± 7.5 vs. 23.4 ± 7.5; SMD 0.123) and eosinophil percentages (1.61 ± 1.62 vs. 1.45 ± 1.27; SMD 0.109).

Modest but statistically significant differences were also observed in selected metabolism-related markers, including lower transferrin (300 ± 53 vs. 306 ± 56; SMD −0.106) and folic acid levels (8.52 ± 4.62 vs. 9.06 ± 4.83; SMD −0.115), and higher vitamin B12 concentrations (316 ± 140 vs. 301 ± 134; SMD 0.109). CMV IgG titers were slightly higher among seropositive individuals (117 ± 92 vs. 106 ± 91; SMD 0.129).

### 3.4. Mortality and Morbidity Associations Screening

The mean follow-up time was 12.7 ± 6.3 years in both groups. Cox proportional hazards models clustered by matched strata and residually adjusted for age at cohort entry, sex, and five-level socioeconomic status were used to evaluate associations with all-cause mortality and incident morbidity.

**Figure 2:**
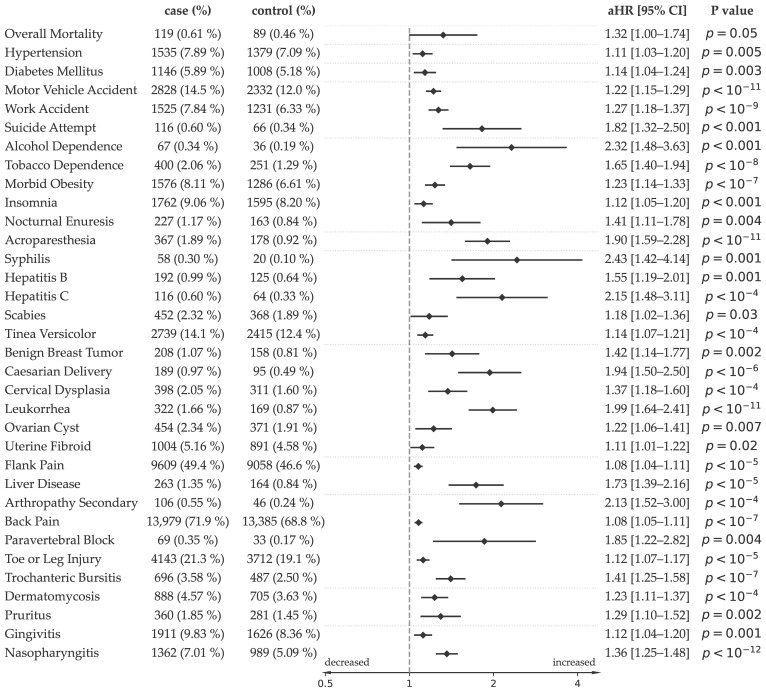
Associations between *Toxoplasma* seropositivity and incident health outcomes. Forest plot displaying adjusted hazard ratios (aHRs) and 95% confidence intervals for incident outcomes during follow-up among seropositive individuals compared with matched persistently seronegative controls. Effect estimates were derived from Cox proportional hazards models accounting for the matched cohort design and residually adjusted for age at cohort entry, sex, and 5-level socioeconomic status. Outcomes shown remained statistically significant after false discovery rate correction in the predefined screening of approximately 300 medical conditions. The vertical dashed line represents the null value (HR = 1). Points to the right of 1 indicate increased risk among seropositive individuals. Case and control event counts with percentages are shown on the left; adjusted hazard ratios and *p* values are displayed on the right.

A borderline increase in overall mortality was observed among seropositive individuals (119 [0.61%] vs. 89 [0.46%]; adjusted HR 1.32 [1.00–1.74], *p* = 0.05). Among the 300 predefined medical conditions screened, multiple statistically significant associations were observed after false discovery rate correction. Effect estimates derived from the matched Cox models are presented in Figure 2.

The largest effect sizes were observed for behavioral and accident-related outcomes. Seropositive individuals demonstrated increased rates of tobacco dependence (aHR 1.65 [1.40–1.94], *p* < 10^−8^), alcohol dependence (2.32 [1.48–3.63], *p* < 0.001), suicide attempt (1.82 [1.32–2.50], *p* < 0.001), work accidents (1.27 [1.18–1.37], *p* < 10^−9^), and motor vehicle accidents (1.22 [1.15–1.29], *p* < 10^−11^). Insomnia (1.12 [1.05–1.20], *p* < 0.001) and nocturnal enuresis (1.41 [1.11–1.78], *p* = 0.004) were also more frequent. Morbid obesity was also increased (1.23 [1.14–1.33], *p* < 10^−7^).

A second cluster involved infectious and sexually transmitted conditions. Increased risks were observed for hepatitis C (2.15 [1.48–3.11], *p* < 10^−4^), hepatitis B (1.55 [1.19–2.01], *p* < 0.001), and syphilis (2.43 [1.42–4.14], *p* = 0.001). Human papillomavirus-related outcomes, including cervical dysplasia (1.37 [1.18–1.60], *p* < 10^−4^), were also enriched. Additional infectious diagnoses included nasopharyngitis (1.36 [1.25–1.48], *p* < 10^−12^), gingivitis (1.12 [1.04–1.20], *p* = 0.001), dermatomycosis (1.23 [1.11–1.37], *p* < 10^−4^), and tinea versicolor (1.14 [1.07–1.21], *p* < 10^−4^). Liver disease was also more frequent among seropositive individuals (1.73 [1.39–2.16], *p* < 10^−5^).

Several gynecologic and reproductive diagnoses were increased, including leukorrhea (1.99 [1.64–2.41], *p* < 10^−11^), ovarian cyst (1.24 [1.07–1.43], *p* = 0.004), and Caesarian delivery (1.94 [1.50–2.50], *p* < 10^−6^). Benign breast tumors were modestly but significantly increased (1.42 [1.14–1.77], *p* = 0.002).

A few musculoskeletal and pain-related diagnoses were enriched, back pain was slightly more frequent (1.08 [1.05–1.10], *p* < 10^−7^), and significantly more individuals underwent paravertebral block procedures (1.85 [1.22–2.82], *p* = 0.04), suggesting increased interventional pain management. Specific rare pathologies appeared to be more frequent, notably acroparesthesia (1.90 [1.59–2.28], *p* < 10^−11^), trochanteric bursitis (1.41 [1.25–1.58], *p* < 10^−7^), and secondary arthropathy (2.13 [1.52–3.00], *p* < 10^−4^).

### 3.5. Kaplan–Meier Analyses

**Figure 3:**
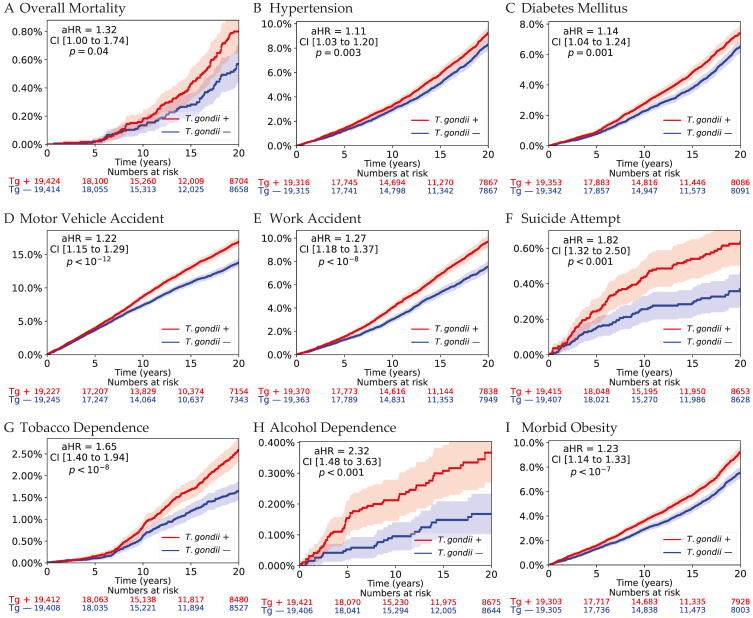
Kaplan–Meier cumulative incidence curves for mortality and factors associated with increased mortality. Kaplan–Meier curves depicting cumulative incidence over up to 20 years of follow-up comparing *Toxoplasma*-seropositive individuals (red) with matched seronegative controls (blue). Shaded areas represent 95% confidence intervals. Hazard ratios (aHRs) and 95% confidence intervals were derived from Cox proportional hazards models accounting for matched strata and adjusted for age at cohort entry, sex, and 5-level socioeconomic status. Numbers at risk at prespecified time points are shown below each panel. Progressive separation of curves over time is observed across outcomes, indicating sustained divergence in cumulative risk.

**Figure 4:**
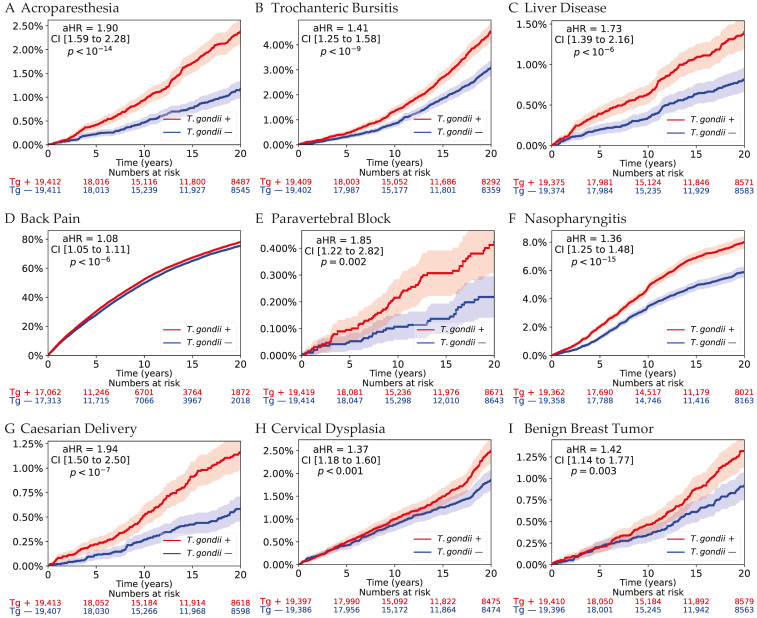
Kaplan–Meier cumulative incidence curves for selected organ system outcomes. Kaplan–Meier curves illustrating cumulative incidence comparing *Toxoplasma*-seropositive individuals (red) with matched seronegative controls (blue). Shaded areas represent 95% confidence intervals. Adjusted hazard ratios were estimated using Cox proportional hazards models stratified by matched pairs and adjusted for age, sex, and socioeconomic status. Numbers at risk are provided beneath each panel. Across multiple domains, seropositive individuals demonstrate higher cumulative incidence with gradual curve divergence over long-term follow-up, consistent with sustained excess risk.

Kaplan–Meier curves for selected outcomes are presented in Figure 3 and Figure 4. For overall mortality (Figure 3A), cumulative incidence curves demonstrated gradual divergence over time, with higher mortality among seropositive individuals. Although absolute differences remained typically small in this relatively young cohort, separation increased progressively during follow-up, consistent with the hazard ratio estimates reported in the Cox models.

For metabolic outcomes, cumulative incidence curves for diabetes mellitus and morbid obesity (Figure 3B,C) showed sustained separation between groups that widened over time.

Behavioral and accident-related outcomes exhibited clear divergence patterns. Motor vehicle accidents and work-related accidents (Figure 3D,E) demonstrated early separation that continued to increase throughout follow-up. Suicide attempts (Figure 3F) showed a similar pattern, with a steeper cumulative incidence trajectory among seropositive individuals.

Substance-related outcomes also displayed progressive divergence. Tobacco dependence and alcohol dependence curves (Figure 3G,I) separated early and continued to diverge across the observation period. Morbid obesity (Figure 3H) likewise demonstrated persistent cumulative differences over time.

Across outcomes, divergence was generally gradual and sustained rather than apparent at the early follow-up period. Adjustment for baseline healthcare utilization, measured as the number of outpatient visits in the year preceding the index date, did not materially change the estimated associations.

### 3.6. Infectious Diseases Laboratory Markers

To complement the diagnostic associations observed for infectious and sexually transmitted conditions, we compared laboratory-confirmed markers of selected chronic infections among individuals who underwent relevant testing during follow-up (Table 3). Analyses were restricted to those tested for each marker.

Among individuals tested for hepatitis C antibodies, seropositive individuals demonstrated higher positivity rates (0.75% vs. 0.39%; OR 1.96 [1.41 to 2.71], *p* < 0.0001). Hepatitis B surface antigen positivity was also more frequent (1.00% vs. 0.68%; OR 1.47 [1.17 to 1.85], *p* = 0.0008). Among those tested for hepatitis B core antibodies, positivity was more common (52.6% vs. 29.2%; OR 2.69 [1.37 to 5.29], *p* = 0.0047), as was HBe antibody positivity (53.9% vs. 37.0%; OR 1.99 [1.32 to 3.00], *p* = 0.0011).

Cervical human papillomavirus (HPV) PCR testing, recently incorporated into routine cervical cancer screening, showed higher positivity among seropositive individuals, including overall HPV detection (8.60% vs. 6.70%; OR 1.31 [1.18 to 1.46], *p* < 0.0001) and high-risk HPV types (6.68% vs. 5.03%; OR 1.35 [1.20 to 1.53], *p* < 0.0001). HSV 1+2 IgG antibodies were also more frequent (80.2% vs. 75.9%; OR 1.28 [1.10 to 1.49], *p* = 0.0014), as were CMV IgG antibodies (93.4% vs. 91.4%; OR 1.33 [1.23 to 1.45], *p* < 0.0001).

The proportion of individuals undergoing testing for these markers was similar between groups (Table 3), reducing the possibility of differential testing intensity. Overall, laboratory-confirmed markers of chronic viral and sexually transmitted infections were more frequently observed among *Toxoplasma*-seropositive individuals.

## 4. Discussion

In this nationwide matched cohort study of young adults (18–45 years) identified through systematic serologic screening, we examined long-term health outcomes associated with asymptomatic *T. gondii* seropositivity using comprehensive electronic health record data. *T. gondii* establishes a chronic infectious state that is highly prevalent worldwide, yet its long-term systemic consequences in immunocompetent individuals remain incompletely defined. Rather than representing a biologically inert residue of prior exposure, persistent infection reflects an active host–pathogen equilibrium sustained by continuous immune pressure and parasite immune evasion mechanisms, including modulation of innate immune signaling pathways and maintenance of bradyzoite tissue cysts [6,27].

Leveraging longitudinal data from a nationwide integrated healthcare organization, we constructed a rigorously matched cohort with tight calendar alignment, balanced observation windows, and excellent baseline comparability across demographic and socioeconomic variables. The cohort consisted predominantly of young, immunocompetent women undergoing routine screening, thereby reducing referral bias and limiting confounding by pre-existing disease. Standardized mean differences for baseline characteristics were uniformly small, and routine laboratory parameters were largely comparable at cohort entry, supporting the interpretation that participants were broadly healthy at baseline.

Despite this apparent baseline health equivalence, seropositive individuals exhibited a consistent pattern of adverse longitudinal outcomes. Cumulative incidence curves showed progressive divergence over time, with a trend toward increased overall mortality in this relatively young cohort. More strikingly, systematic screening across predefined medical conditions revealed clusters of significantly enriched outcomes after correction for multiple testing. Importantly, in the context of a persistent exposure acquired prior to cohort entry, a consistent direction of association across multiple outcomes is not unexpected, as cumulative divergence in health trajectories over time may manifest predominantly in one direction rather than symmetrically around the null.

Behavioral and risk-related conditions emerged prominently. Increased hazards of tobacco dependence, alcohol dependence, suicide attempt, motor vehicle accidents, and occupational injuries formed a coherent behavioral phenotype suggestive of altered risk evaluation or impulse control. These associations were not confined to a single diagnostic domain but spanned addiction, self-harm, and accidental injury, strengthening the internal consistency of the findings.

Morbid obesity and diabetes also demonstrated increased incidence, with Kaplan–Meier curves showing gradual and sustained separation over time. These findings are notable given the minimal differences in baseline anthropomorphic measures and metabolic laboratory markers, including BMI, fasting glucose, hemoglobin A1c, lipid profile, renal function, and hemoglobin. Together, this pattern suggests that seropositive individuals did not enter the cohort with overt systemic disease but may have experienced greater accumulation of cardiometabolic risk factors during follow-up.

### 4.1. Neurobehavioral Associations and a Risk-Related Phenotype

A central finding of this study was the association between *Toxoplasma* seropositivity and a spectrum of behavioral and risk-related outcomes. Seropositive individuals demonstrated increased long-term incidence of motor vehicle accidents (aHR 1.22), work-related accidents (aHR 1.27), suicide attempts (aHR 1.82), tobacco dependence (aHR 1.65), alcohol dependence (aHR 2.32), and insomnia (aHR 1.11). Although individual effect sizes were modest to moderate, the pattern was directionally consistent across multiple domains, reflecting a potential shift in behavioral regulation and risk exposure.

These findings are concordant with prior epidemiologic observations linking *Toxoplasma* seropositivity to traffic accidents and altered reaction time. Flegr and colleagues reported delayed psychomotor performance and increased accident risk among seropositive drivers [20], with higher risks observed in individuals with recent infection or elevated antibody titers. Subsequent analyses have generally supported associations between latent toxoplasmosis and traffic incidents [11,28], as well as suicidal behavior or self-directed violence [21,23], although results have varied across populations.

Associations between *Toxoplasma* infection and suicidal behavior, substance use, and selected psychiatric phenotypes have also been described in independent cohorts [11,13,16,23,29]. While effect sizes vary and causality remains debated, the recurrence of directionally similar findings across geographically distinct populations strengthens the plausibility of a biologically mediated signal rather than isolated confounding.

### 4.2. Translational Parallels from Experimental Models

Experimental models provide a biologically plausible framework for the observed behavioral associations. In rodents, chronic *T. gondii* infection induces reproducible alterations in risk-related behavior, most notably reduced aversion to predator odors, including feline scent, a phenomenon often referred to as “fatal feline attraction” [7,30,31]. Infected rodents exhibit impaired fear processing, altered exploratory behavior, and changes in risk assessment, effects that have been linked to cyst localization within limbic structures and modulation of dopaminergic signaling pathways. These behavioral changes are thought to increase predation probability and thereby facilitate completion of the parasite’s life cycle.

Mechanistic studies have implicated altered dopaminergic signaling, including parasite-encoded tyrosine hydroxylase-like enzymes and changes in host dopamine metabolism. Chronic neuroinflammation, microglial activation, and synaptic remodeling have also been documented in experimental models. These biological alterations converge on neural circuits involved in reward processing, fear conditioning, and behavioral inhibition [6,27].

### 4.3. Risk-Taking as a Behavioral Continuum

While extrapolation from rodents to humans must be approached cautiously, the cross-species consistency in neural tropism and encystment biology of *T. gondii* is notable. *T. gondii* forms persistent tissue cysts in neural tissue in both rodents and humans, and experimental data suggest that chronic infection may influence dopaminergic signaling, neurotransmitter systems, and neuroinflammatory pathways. Human neuroimaging and seroepidemiologic studies have reported associations between seropositivity and altered reaction time, personality traits, and risk-related behaviors. The convergence of experimental animal data and human epidemiologic observations supports the hypothesis that subtle parasite-associated modulation of neural circuits involved in risk evaluation and impulse control could contribute to the behavioral phenotype observed in this study.

Higher *T. gondii* seroprevalence has also been reported in several neuropsychiatric and neurocognitive conditions, including schizophrenia and cognitive decline. Moreover, recent retrospective pharmacoepidemiologic analyses have suggested that exposure to anti–*Toxoplasma*-active agents may be associated with reduced incidence of selected neuropsychiatric disorders [16,17,18,32].

The convergence between altered predator avoidance in rodents and increased accident risk and addiction-related outcomes in humans is compatible with a host–parasite interaction model in which chronic infection subtly modulates neural regulatory systems. Such modulation, even if quantitatively small at the individual level, may have detectable epidemiologic consequences in large populations. In this study of otherwise healthy individuals, the effect sizes are modest and do not imply deterministic behavioral change. However, the gradual divergence observed in the Kaplan–Meier curves is consistent with small shifts in population-level risk distribution that, over extended follow-up, translate into measurable differences in accidents, substance use, and self-harm outcomes, even in the absence of overt neuropsychiatric disease.

### 4.4. Clustering with Infectious Diseases and Sexually Transmitted Infections

Beyond neurobehavioral outcomes, *T. gondii* seropositivity was associated with increased incidence of multiple infectious conditions, including hepatitis B, hepatitis C, syphilis, HPV-related disease, dermatomycosis, nasopharyngitis, scabies, and leukorrhea. Laboratory-confirmed markers of chronic infection were also more prevalent among those tested, including hepatitis C antibodies, hepatitis B surface antigen, HPV PCR positivity, HSV IgG, and CMV IgG.

The magnitude of association for several sexually transmitted infections, particularly syphilis (aHR 2.52) and hepatitis C (OR 2.17), suggests clustering within a broader exposure or vulnerability phenotype. While coinfection patterns in specific demographic subgroups may partially contribute, the consistency across both diagnostic codes and laboratory-confirmed markers supports the consideration of several non-mutually exclusive mechanisms.

#### 4.4.1. Behavioral Mediation

A first potential explanation is behavioral mediation. The same risk-related phenotype associated with accidents, substance dependence, and self-harm may extend to sexual risk behaviors, including higher partner turnover or reduced protective practices. Under this framework, increased sexually transmitted infections (STIs) incidence would represent a downstream behavioral consequence rather than a direct biological effect of the parasite. Although sexual behavior cannot be directly assessed in EHR data, the co-occurrence of substance dependence, accident risk, and STI diagnoses is compatible with a shared behavioral substrate.

#### 4.4.2. Immune Modulation

An alternative, not mutually exclusive explanation involves immune modulation, either as a consequence of chronic *T. gondii* infection or reflecting a pre-existing immune susceptibility that facilitated persistent infection in the first place. Chronic *T. gondii* carriage is characterized by sustained Th1-polarized immune activation, ongoing interferon-gamma signaling, and lifelong tissue cyst persistence. Even in immunocompetent hosts, such chronic immune engagement may influence immune homeostasis over time [27].

At baseline, we observed small but directionally consistent differences in leukocyte differentials and selected micronutrient markers, including lower neutrophil proportions, modest shifts in lymphocyte and eosinophil fractions, and differences in transferrin, folate, vitamin B12, and CMV IgG titers. Although effect sizes were modest and values largely remained within conventional reference ranges, the coherence across immune-related parameters suggests possible immunologic divergence in seropositive individuals.

These findings are compatible with chronic low-grade immune modulation or altered immune set-point, which could influence susceptibility to secondary infections or inflammatory conditions at the population level. Whether such shifts are causally induced by *T. gondii* or reflect host characteristics that predispose to both persistent toxoplasmosis and other infections cannot be determined in the present observational framework and warrants targeted mechanistic investigation.

#### 4.4.3. Alternative Transmission Routes

A third hypothesis concerns alternative transmission routes. In humans, ingestion of oocysts from the environment or tissue cysts in undercooked meat remains the established mode of transmission. However, experimental data in multiple non-human species demonstrate reproductive tissue invasion and parasite presence in semen. In a recent human study of men attending a fertility clinic, Tong et al. identified *T. gondii* tissue cysts in semen samples of seropositive individuals. Initial slide examination detected cysts in 35 of 50 seropositive men, and upon successive examination of additional stained slides, cysts were identified in all 50 seropositive individuals. In situ hybridization confirmed bradyzoite-specific BAG1 mRNA within the intra-cyst structures, supporting the identification of *T. gondii* bradyzoites [33].

Epidemiologic data further reinforce biological plausibility. In a study of couples attending an assisted reproduction center, female seropositivity was significantly more frequent when the male partner was seropositive, whereas male seropositivity was not associated with female partner status (prevalence ratio 1.42 for the male-to-female direction) [34]. This asymmetric association is not fully explained by shared environmental exposures and is compatible with the possibility of male-to-female transmission, although residual confounding cannot be excluded.

While transmission efficiency and population-level contribution of *T. gondii* cysts identifiable in human semen remain to be quantified, the co-clustering of *Toxoplasma* seropositivity with multiple sexually transmitted infections observed in our cohort, together with accumulating experimental and epidemiologic evidence, supports consideration of sexual transmission as a potential route, though currently unproven route, warranting further targeted investigation.

### 4.5. Mortality Signal

Although the cohort was predominantly young and healthy, we observed a borderline increase in all-cause mortality among seropositive individuals, with divergence in cumulative incidence curves over time. Given the age distribution and modest effect size, this finding should be interpreted cautiously. However, it raises the possibility that the slight but consistent shift in comorbidity profile observed during follow-up may accumulate longitudinally and may translate into increased mortality in the long run.

### 4.6. Strengths and Limitations

This study has several important strengths. First, it was conducted within a large, nationwide health maintenance organization with longitudinal electronic health records spanning more than two decades, enabling comprehensive capture of diagnoses, laboratory measurements, medication use, and mortality. The matched cohort design incorporated strict 1:1 matching without replacement, tight calendar alignment of index dates, and balanced observation windows, thereby minimizing immortal time bias and secular trend confounding. Post-matching standardized mean differences demonstrated excellent baseline balance across demographic and socioeconomic variables, with strong temporal alignment. Time-to-event analyses using Cox proportional hazards models accounted for matched strata and residual confounding by age, sex, and socioeconomic status. In addition, systematic screening of approximately 300 predefined medical conditions reduced the risk of selective reporting and allowed evaluation across broad health domains rather than isolated hypothesis-driven outcomes.

Several limitations should be acknowledged. As an observational study, causal inference cannot be definitively established, and residual confounding by unmeasured behavioral, environmental, or lifestyle factors cannot be entirely excluded. However, the convergence of associations across behavioral, infectious, metabolic, and procedural domains reduces the likelihood that a single unmeasured factor fully explains the observed multi-domain pattern. The longitudinal design, with exclusion of individuals with clinical toxoplasmosis and restriction to a young, largely healthy screening population, reduces but does not eliminate the possibility that pre-existing behavioral differences contributed to exposure. Because *T. gondii* infection is typically acquired years before serologic testing in adulthood, baseline behavioral differences at the time of testing are unlikely to fully account for the progressive divergence in outcomes observed during longitudinal follow-up. Although some outcomes may be influenced by healthcare utilization patterns, the strict alignment of demographic characteristics, calendar time, entry into the electronic health record system, and duration of follow-up between matched individuals reduces the likelihood that healthcare utilization differences alone explain the findings. Consistently, additional adjustment for baseline healthcare utilization did not materially alter the observed associations, further reducing the likelihood that differential healthcare contact alone explains the results. Moreover, population-based analyses have shown that while environmental and behavioral exposures such as dietary habits or animal contact may contribute to infection risk, demographic and socioeconomic factors account for a substantial proportion of observed seroprevalence variation; these factors were carefully matched in our cohort [4].

Because the cohort predominantly consisted of women undergoing routine reproductive screening, the findings primarily apply to young women. Although this limits direct extrapolation of effect sizes to male populations, the biological mechanisms discussed, including neural encystment, immune modulation, and chronic host–parasite interaction, are not known to be sex-specific. Future studies in cohorts with a higher proportion of men may help confirm whether similar risk patterns are observed across sexes.

It should be noted that seropositivity reflects prior exposure but does not provide information on the timing of infection, parasite burden, strain variation, or tissue localization, limiting mechanistic interpretation. Because *T. gondii* infection is most commonly acquired during childhood or adolescence, long before routine serologic testing in adulthood, substance dependence or metabolic differences recorded in adult electronic health records are unlikely to represent baseline determinants of infection risk. Accordingly, differences in these variables at the time of serologic testing are more likely to represent downstream consequences of infection-related behavioral or metabolic effects rather than baseline determinants of exposure. For this reason, in contrast to demographic and temporal variables, which were tightly matched in our cohort, behavioral or metabolic variables such as smoking status, alcohol consumption, or body mass index were not included in the matching algorithm. Adjusting for such variables could therefore introduce overadjustment bias if these behaviors lie on the causal pathway between infection and subsequent health outcomes. In this cohort, *Toxoplasma* screening was largely performed as part of routine obstetric care and was not triggered by smoking status, BMI, or other lifestyle characteristics, making confounding through the testing process less likely. Nevertheless, residual confounding related to unmeasured behavioral or lifestyle characteristics cannot be completely excluded.

Notwithstanding these limitations, large-scale longitudinal EHR-based analyses represent one of the most informative currently available approaches for evaluating population-level associations of chronic, often asymptomatic infections. By combining strict matching procedures, calendar alignment, and comprehensive outcome assessment, the present study implemented robust safeguards against major sources of bias within an observational framework.

### 4.7. Implications

The convergence of behavioral risk amplification, clustering with selected infectious conditions, cardiometabolic risk accumulation, and a trend toward increased mortality raises the possibility of a downstream health trajectory characterized by incremental risk accumulation rather than an immediate pathogenic effect. Importantly, these associations were observed in a population without clinical toxoplasmosis and with largely normal laboratory profiles at study entry, emphasizing that the exposure of interest represents latent, asymptomatic seropositivity.

Taken together, the findings suggest that asymptomatic *T. gondii* seropositivity in young adults may be associated with subtle but longitudinally meaningful differences in health trajectories. These differences include increased risk-taking behaviors, greater incidence of selected infectious conditions including sexually transmitted infections, accumulation of cardiometabolic risk factors, and a potential early signal of increased mortality.

These observations are consistent with an evolutionary framework of delayed pathogenicity in *Toxoplasma gondii*, a parasite that infects a wide range of species but can reproduce sexually only in felids following predation of its host. In this framework, the parasite may benefit from progressive weakening of host fitness by various means, in a manner that could facilitate predation, while limiting impairments at younger ages so as not to endanger host reproduction and population continuity [35].

Given the high global prevalence of latent *T. gondii* infection, even modest shifts in behavioral or infectious risk distributions could translate into meaningful population-level consequences in the long run. These findings provide population-level phenotypic evidence consistent with the biological relevance of persistent bradyzoite-stage infection in immunocompetent hosts and reinforce the broader concept that parasitic interactions may extend beyond immediate infectious sequelae to influence long-term health trajectories.

Future studies integrating parasite burden quantification, tissue localization, strain characterization, and immunophenotyping, possibly using advanced imaging and molecular testing techniques, may help clarify underlying mechanisms. Ultimately, interventional studies targeting latent infection would be necessary to determine whether reducing persistent parasite burden alters long-term outcomes.

## 5. Conclusions

In a large cohort of predominantly young, asymptomatic adults identified through systematic screening and followed for more than twelve years, *Toxoplasma gondii* IgG seropositivity was associated with a consistent pattern of differences across multiple health domains, including increased incidence of risk-related behaviors, infectious conditions including sexually transmitted infections, immune-related laboratory differences, and a trend toward increased mortality. These findings suggest that latent *T. gondii* infection may be associated with measurable long-term differences across behavioral, metabolic, and infectious domains, although causal mechanisms cannot be established from this observational study.

## Figures and Tables

**Table 1:** Baseline characteristics of matched seropositive and persistently seronegative individuals.

		Case	Control	SMD
N		19,443	19,443	
sex	Female	18,796 (96.7%)	18,796 (96.7%)	0.000
	Male	647 (3.33%)	647 (3.33%)	0.000
index test year		2013 ± 6	2013 ± 6	−0.016
age category (at index date)	18–29	10,630 (54.7%)	10,600 (54.5%)	−0.004
	30–39	7607 (39.1%)	7661 (39.4%)	0.003
	40–45	1206 (6.20%)	1182 (6.08%)	−0.006
year of first EHR record		2006 ± 5	2006 ± 5	0.005
BMI (kg/m^2^)		25.11 ± 5.48	25.04 ± 5.26	0.002
	missing	897 (4.61%)	998 (5.13%)	0.013
BP systolic (mmHg)		111.0 ± 12.3	110.6 ± 12.2	0.033
	missing	207 (1.06%)	227 (1.17%)	−0.010
Ethnic sector	Arab	7292 (37.5%)	7292 (37.5%)	0.000
	General	9599 (49.4%)	9599 (49.4%)	0.000
	Jewish Ultra-orthodox	2552 (13.1%)	2552 (13.1%)	0.000
Smoking status	Non-smoker	14,944 (84.6%)	15,439 (87.1%)	−0.073
	Past smoker	183 (1.04%)	179 (1.01%)	0.003
	Smoker	2539 (14.4%)	2102 (11.9%)	0.074
	missing	1777 (9.14%)	1723 (8.86%)	0.010
Socio-economic status	(1–20)	7.849 ± 3.556	7.985 ± 3.486	−0.038

Baseline demographic and clinical characteristics of matched *Toxoplasma gondii* seropositive individuals (cases) and persistently seronegative controls. Continuous variables are presented as mean ± standard deviation (SD) and categorical variables as number (%). Standardized mean differences (SMD) were calculated to assess covariate balance between groups; absolute SMD values < 0.05 indicate adequate balance. The index date corresponds to the date of the first recorded *T. gondii* serologic test. Missing values are shown as explicit categories where applicable. Abbreviations: BMI, body mass index; BP, blood pressure; EHR, electronic health record; SMD, standardized mean difference; SD, standard deviation.

**Table 2:** Laboratory results shift in seropositive patients.

Lab Test	Case		Control		SMD	*p* Value
	Tested	Mean ± SD	Tested	Mean ± SD		
White Blood Cells	19,299	8.51 ± 2.49	19,317	8.78 ± 2.45	−0.107	3.5 × 10^−32^
Neutrophils %	19,240	66.5 ± 8.8	19,270	67.7 ± 8.7	−0.136	9.5 × 10^−41^
Lymphocytes %	19,240	24.3 ± 7.5	19,270	23.4 ± 7.5	0.123	1.2 × 10^−36^
Eosinophils %	19,240	1.61 ± 1.62	19,270	1.45 ± 1.27	0.109	1.6 × 10^−25^
Transferrin	9566	300 ± 53	10,395	306 ± 56	−0.106	4.4 × 10^−11^
Folic Acid	10,090	8.52 ± 4.62	11,071	9.06 ± 4.83	−0.115	1.4 × 10^−15^
Vitamin B12	14,887	316 ± 140	15,658	301 ± 134	0.109	8.7 × 10^−23^
CMV Ab IgG	15,420	117 ± 92	15,771	106 ± 91	0.129	4.4 × 10^−35^

Baseline laboratory measurements obtained before the index date for *T. gondii* seropositive individuals (cases) and matched seronegative controls. Values are presented as mean ± standard deviation (SD). “Tested” indicates the number of individuals with an available laboratory measurement. Standardized mean differences (SMD) quantify effect size between groups. *p* values were calculated using two-sample comparisons. Abbreviations: SD, standard deviation; SMD, standardized mean difference; CMV, cytomegalovirus.

**Table 3:** Prevalence of selected chronic viral and sexually transmitted infections among individuals tested during follow-up.

Lab Test	Case		Control		OR (CI)	*p* Value
	Tested	Positive	Tested	Positive		
Hepatitis C Ab	14,078	106 (0.75%)	14,234	55 (0.39%)	1.96 [1.41 to 2.71]	<0.0001
HB Surface Ag	18,538	185 (1.00%)	18,678	127 (0.68%)	1.47 [1.17 to 1.85]	0.0008
HB Core Ab IgG&IgM	78	41 (52.6%)	72	21 (29.2%)	2.69 [1.37 to 5.29]	0.0047
HBe Ab	217	117 (53.9%)	173	64 (37.0%)	1.99 [1.32 to 3.00]	0.0011
HPV PCR	9507	818 (8.60%)	9639	646 (6.70%)	1.31 [1.18 to 1.46]	<0.0001
Other High Risk HPV	9507	635 (6.68%)	9639	485 (5.03%)	1.35 [1.20 to 1.53]	<0.0001
HSV (1+2) IgG Ab	1900	1523 (80.2%)	2024	1536 (75.9%)	1.28 [1.10 to 1.49]	0.0014
CMV Ab IgG	15,941	14,895 (93.4%)	16,369	14,969 (91.4%)	1.33 [1.23 to 1.45]	<0.0001

Proportion of positive laboratory results among individuals tested during follow-up after the index date. For each infection, the number tested and the number (%) with a positive result are shown for both groups. Odds ratios (OR) with 95% confidence intervals (CI) compare seropositive individuals with matched seronegative controls among those tested. Some assays (e.g., hepatitis B core antibody, HBe antibody, and HPV genotyping) represent reflex tests performed conditional on prior screening results. Results reflect infections detected during longitudinal follow-up and do not establish temporality or causality. Abbreviations: OR, odds ratio; CI, confidence interval; HPV, human papillomavirus; HSV, herpes simplex virus; CMV, cytomegalovirus; HB, hepatitis B; PCR, polymerase chain reaction.

## Data Availability

The data presented in this study are available on request from the corresponding author due to national health privacy regulations.

## Abbreviations

aHR	adjusted Hazard Ratio
BMI	Body mass Index
CI	Confidence Interval
CMV	Cytomegalovirus
EHRs	electronic health records
HR	Hazard ratios
HSV	Herpes Simplex Virus
HPV	Human Papillomavirus
ICD	International Classification of Diseases
LHS	Leumit Health Services
SD	Standard Deviation
SMD	standardized mean differences
STI	sexually transmitted infections
